# (Dis)Solving the problem of aberrant protein states

**DOI:** 10.1242/dmm.048983

**Published:** 2021-05-04

**Authors:** Charlotte M. Fare, James Shorter

**Affiliations:** 1 Department of Biochemistry and Biophysics, Perelman School of Medicine at the University of Pennsylvania, Philadelphia, PA 19104, USA; 2 Biochemistry and Molecular Biophysics Graduate Group, Perelman School of Medicine at the University of Pennsylvania, Philadelphia, PA 19104, USA

**Keywords:** Disaggregase, Neurodegeneration, Phase transition

## Abstract

Neurodegenerative diseases and other protein-misfolding disorders represent a longstanding biomedical challenge, and effective therapies remain largely elusive. This failure is due, in part, to the recalcitrant and diverse nature of misfolded protein conformers. Recent work has uncovered that many aggregation-prone proteins can also undergo liquid–liquid phase separation, a process by which macromolecules self-associate to form dense condensates with liquid properties that are compositionally distinct from the bulk cellular milieu. Efforts to combat diseases caused by toxic protein states focus on exploiting or enhancing the proteostasis machinery to prevent and reverse pathological protein conformations. Here, we discuss recent advances in elucidating and engineering therapeutic agents to combat the diverse aberrant protein states that underlie protein-misfolding disorders.

## Abstract (precis)

**Summary:** This Special Article discusses cutting-edge approaches to counter aberrant phase transitions in disease using protein disaggregase- and RNA-based strategies.

## Introduction

Protein-misfolding diseases are a pernicious public health problem. Errors in protein folding drive neurodegenerative disorders, such as Alzheimer's disease (AD), Huntington's disease (HD), Parkinson's disease (PD), amyotrophic lateral sclerosis (ALS) and frontotemporal dementia (FTD), as well as other diseases such as cancer and type 2 diabetes (Chuang et al., 2018). Cells are equipped with mechanisms to combat aberrations in protein folding, but these systems can become overwhelmed or dysfunctional. Therefore, one strategy to confront diseases caused by pathological protein states is to bolster the innate proteostasis machinery (Mack and Shorter, 2016).

**Fig. 1.**
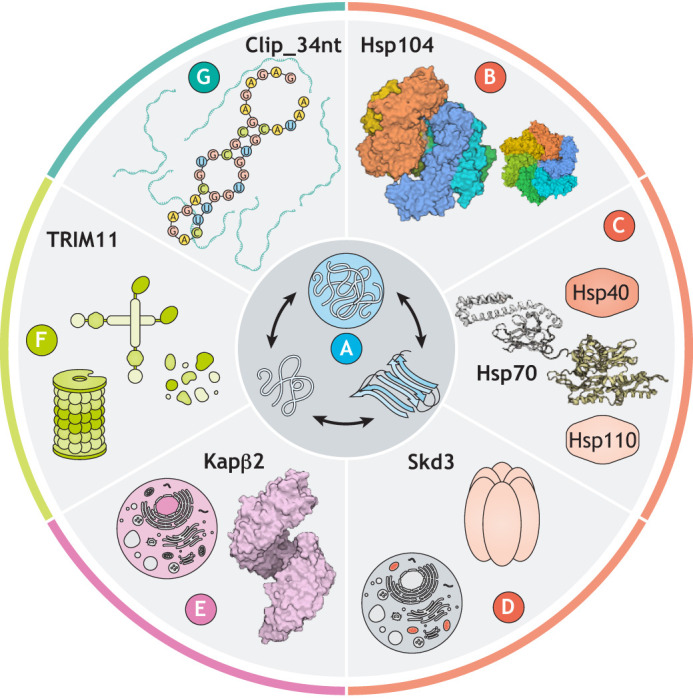
**Protein disaggregases and bait RNAs to counter aberrant protein states.** (A) Native proteins (left) can transition to pathological states, which can present initially as liquid–liquid phase-separated condensates (top) or as ordered amyloids (right). To prevent and reverse these disease-causing processes, researchers are working to understand how protein disaggregases can be harnessed against diseases that remain incurable. (B-D) Three protein-disaggregase systems, Hsp104 [B; Protein Data Bank (PDB) 5VJH and 5VYA]; Hsp110, Hsp70 (PDB 2KHO) and Hsp40 (C); and Skd3 (D) all use ATP hydrolysis to power protein disaggregation. (E) Kapβ2 (PDB 4FDD) is a nuclear-import receptor that acts as a disaggregase in an ATP-independent manner. (F) TRIM family proteins, including TRIM11 and TRIM19, also act as ATP-independent disaggregases in concert with the proteosome to degrade aberrant proteins. (G) Bait RNAs such as Clip_34nt can also act as chaperones by preventing aberrant phase transitions of TDP-43.

Protein folding is a thermodynamically driven process by which the primary amino-acid sequence of a polypeptide is arranged in three-dimensional space to adopt a specific structure (Englander and Mayne, 2017). However, in the crowded environment of the cell, protein folding often relies on protein chaperones to ensure conformational and functional integrity (Balchin et al., 2020; Rothman and Schekman, 2011). The process of protein folding can go awry due to many factors, including mutations, environmental stress and aging (Jarosz et al., 2010). Indeed, the issue of protein misfolding is highly diverse. Each protein-misfolding disease is associated with different proteins and distinct protein conformations (Kelly, 2021). Some diseases are connected to the formation of stable cross-β fibrils termed amyloids (Fig. 1A) (Chuang et al., 2018). Cross-β fibrils of amyloid beta (Aβ) and tau are associated with AD, amyloid inclusions of α-synuclein are associated with PD, and amyloid forms of huntingtin with an expanded polyglutamine (polyQ) tract are connected to HD (Chuang et al., 2018). Although the amyloids found in each of these diseases share a common general cross-β structure, they differ in which cell types they affect, as well as their subcellular localization and what cellular processes they impinge upon (Chuang et al., 2018). This situation is further complicated by the fact that the precise intermolecular contacts within a cross-β amyloid can vary, leading to distinct polymorphs or ‘strains’ with unique properties (Shorter, 2010). As such, there may not be a simple panacea for all amyloidoses and combination therapies may be required (Shorter, 2010).

Other neurodegenerative diseases, such as ALS and FTD, also show heterogeneous patterns of aberrant protein states. Several of the proteins associated with ALS/FTD, including TDP-43 (also known as TARDBP), FUS, TAF15, EWSR1, HNRNPA1 and HNRNPA2, are RNA-binding proteins (RBPs) that also contain a prion-like domain (PrLD) (Cushman et al., 2010; Harrison and Shorter, 2017; King et al., 2012). These proteins play a critical role in RNA metabolism, and, during times of stress, e.g. heat, osmotic or oxidative stress, many RBPs with PrLDs are exported from the nucleus to the cytoplasm, where they condense to store RNA in liquid–liquid phase-separated condensates called stress granules (SGs) (Harrison and Shorter, 2017; Li et al., 2013). SGs are formed by proteins and RNAs making many multivalent intramolecular contacts, and, when dysregulated, some aggregation-prone components of these condensates can mature into gel-like and solid inclusions (Boeynaems et al., 2018; McGurk et al., 2018; Zhang et al., 2019). The process of an aberrant liquid-to-solid phase transition is suggested to underlie the appearance of the cytoplasmic depositions observed in patient tissues (Zhang et al., 2019). Thus, any potential therapeutic approaches for ALS and FTD will need to account for a range of harmful protein conformations that arise throughout the progression of disease, while simultaneously maintaining functional protein assemblies (Fig. 1A) (Portz et al., 2021).

It has long been suggested that microscopic liquid–liquid phase separation is an early step in the progression toward amyloid formation (Serio et al., 2000). Indeed, it is now clear that amyloid-like states can emerge from liquid condensates (Fig. 1A) (Molliex et al., 2015; Patel et al., 2015). Phase separation and the eventual energetic collapse of condensed proteins into an amyloid state may therefore represent two points on a continuum of potential conformations a protein can adopt. It is not always clear whether the liquid, gel or solid states are the most toxic for any given protein (Portz et al., 2021). Thus, strategies to counteract proteotoxicity will need to consider the physical and chemical properties of precise toxic states. For example, in yeast and in various neuronal models, liquid forms of TDP-43 can be toxic (Bolognesi et al., 2019; Gasset-Rosa et al., 2019), whereas amyloid-like forms can be functional, as in the case of myogranules in muscle (Vogler et al., 2018). However, in other contexts, liquid forms of TDP-43 can be functional, allowing the protein to perform its normal pre-mRNA splicing activity (Conicella et al., 2020), and brain-derived solid-phase TDP-43 assemblies can be neurotoxic (Laferriere et al., 2019). Accordingly, therapeutic strategies aimed at disrupting specific condensates must take context into account. Ideally, therapeutic agents would preserve the functional condensates and eliminate deleterious ones.

To study protein-misfolding diseases, researchers have employed numerous model systems, including *in vitro* biochemical assays, as well as yeast, fly, worm and mammalian models (Gitler et al., 2017). Here, we outline how research using these models has led to some of the important advances toward developing strategies to treat diseases caused by aberrant protein states.

## Hsp104

Hsp104 (Fig. 1B) is a yeast protein disaggregase that is a member of the AAA+ (ATPases associated with diverse cellular activities) protein family (Seraphim and Houry, 2020). Hsp104 uses the energy from adenosine triphosphate (ATP) binding and hydrolysis to remodel and disaggregate both ordered amyloids and disordered protein aggregates (Shorter and Southworth, 2019; Ye et al., 2020). Animals, though, do not have an exact Hsp104 homolog, and so deploying Hsp104 when endogenous chaperones fail could be an attractive therapeutic approach (Cushman-Nick et al., 2013; Lo Bianco et al., 2008; Perrin et al., 2007; Shorter, 2008). Hsp104 is a powerful protein disaggregase, but its activity against human neurodegenerative disease-associated proteins can be limited (DeSantis et al., 2012; Jackrel et al., 2014). Thus, potentiated Hsp104 variants have been generated by engineering an autoregulatory domain (Jackrel and Shorter, 2015). These enhanced Hsp104 variants can rescue toxicity caused by α-synuclein, FUS and TDP-43 in yeast, *Caenorhabditis* *elegans* and mammalian cell model systems (Jackrel et al., 2014; Mack et al., 2020 preprint; March et al., 2020; Yasuda et al., 2017).

However, there are several barriers to introducing potentiated Hsp104 variants as therapeutic agents into patients (Shorter, 2008; Tariq et al., 2019). One issue is off-target effects. Hsp104 is a general protein disaggregase, and thus deregulating Hsp104 activity may create some risk for off-target unfolding of natively folded substrates. Indeed, the first generation of potentiated Hsp104 variants was effective in disease models due to increased ATPase activity and enhanced disaggregase activity, but in some circumstances could also be toxic in the absence of any disease substrate, suggesting that enhanced activity could lead to unfolding of non-pathogenic bystander proteins (Jackrel et al., 2014; Jackrel and Shorter, 2014). More recently, our group has engineered the AAA+ domains of Hsp104 to develop specific variants that can rescue the toxicity caused by proteins associated with neurodegeneration (Tariq et al., 2019). These AAA+-targeted Hsp104 variants are also more active ATPases, disaggregases and refoldases, but were not themselves toxic (Tariq et al., 2019).

We anticipate that further tuning Hsp104 disaggregase activity might also be beneficial in targeting specific diseases. For example, specific mutations to the substrate-binding tyrosine residues in the interior channel of Hsp104 produces variants that act on α-synuclein, but not on FUS or TDP-43 (Mack et al., 2020 preprint). Intriguingly, some of the α-synuclein-specific Hsp104 variants rescue α-synuclein toxicity without dissolving cytoplasmic inclusions, raising the possibility that Hsp104 can rescue α-synuclein toxicity by remodeling protein inclusions without disaggregating them (Mack et al., 2020 preprint). Tuning Hsp104 variants to target specific toxic substrates based on their sequence, structure or material properties is an exciting option for treating diseases caused by protein misfolding.

Our group has also recently described natural Hsp104 variants that rescue the toxicity of α-synuclein and TDP-43 in an ATPase-independent manner (March et al., 2020). In this study, March et al. surveyed the natural sequence space of homologous Hsp104 proteins and found that Hsp104 proteins from diverse species can selectively chaperone α-synuclein in yeast and *C. elegans*, or TDP-43 in yeast and human cells, without off-target toxicity (March et al., 2020). These naturally enhanced variants did not display enhanced disaggregase activity *in vitro*, nor did they stringently require ATP binding and hydrolysis to rescue proteotoxicity in yeast. Rather, they passively inhibited the aggregation of specific substrates (March et al., 2020). Hsp104 variants that do not require excessive ATPase activity for potentiated disaggregase activity are an exciting prospect, as they would be less energetically taxing on degenerating neurons that may already be afflicted by ATP depletion. Further characterization of this passive Hsp104 activity may therefore empower us to engineer more sophisticated disaggregases that are well suited for therapeutic applications. It will be of great interest to advance our latest enhanced Hsp104 variants into more complex models of neurodegeneration.

## Hsp110, Hsp70 and Hsp40

Although humans lack an exact Hsp104 homolog, we do have an effective protein-disaggregase system comprised of three components: (1) a member of the Hsp110 (also known as HSPH) family, which acts as both a nucleotide-exchange factor for Hsp70 and exhibits chaperone activity on its own; (2) a member of the Hsp70 (also known as HSPA) family, which binds to the substrate and hydrolyzes ATP; and (3) a member of the Hsp40 (also known as DNAJ) family, which acts as an essential co-chaperone that interacts with Hsp110, Hsp70 and substrate (Fig. 1C). *In vitro*, Hsp110, Hsp70 and Hsp40 cooperate to disaggregate misfolded proteins (Chuang et al., 2018; Duennwald et al., 2012; Shorter, 2011; Torrente and Shorter, 2013; Wentink et al., 2020), and elevated levels of isolated components of the system can be neuroprotective *in vivo* (Bonini, 2002; Taguchi et al., 2019). However, many mechanistic details of the Hsp110–Hsp70–Hsp40 system remain incompletely understood.

To achieve a global understanding of the Hsp70 interactome, Ryu and colleagues used proximity-based approaches to catalog the interaction partners for Hsp70 and Hsc70 (also known as HSPA8), a constitutively expressed Hsp70 family member, in human cells (Ryu et al., 2020). In addition to interacting with co-chaperones, Hsp70 and Hsc70 exhibited a preference for newly translated proteins and ‘orphan’ proteins, which are monomeric proteins that require binding to other proteins for stability and function. Such a preference confirmed that the Hsp70 family plays an essential role in nascent polypeptide folding and protein complex formation (Ryu et al., 2020). Intriguingly, expression of a misfolded protein such as ALS-linked SOD1 shifted the interactome for Hsp70 and Hsc70, with Hsc70 showing more significant engagement with disordered proteins upon SOD1 expression (Ryu et al., 2020). Thus, Hsp70 and Hsc70 are general folding chaperones and also function as part of a disaggregase system to reverse protein aggregation.

Further evidence for the role of Hsp70 in managing disease-relevant disordered proteins *in vivo* comes from studies that identify the nucleolus as a site of Hsp70 activity (Frottin et al., 2019). The nucleolus is a nuclear phase-separated membraneless organelle, and is the site of ribosome biogenesis (Frottin et al., 2019). When the cell encounters environmental stress, misfolded nuclear proteins accumulate in the nucleolus, along with Hsp70 and other chaperones (Frottin et al., 2019). Normally, the nucleolus exhibits very dynamic, liquid-like behavior. However, upon heat shock, the misfolded proteins in the nucleolus, as well as resident nucleolar proteins, show decreased mobility (Frottin et al., 2019). This decreased mobility may be due to aberrant interactions between misfolded polypeptides and nucleolar residents (Frottin et al., 2019). The dynamic behavior of nucleolar proteins cannot be recovered when Hsp70 is inhibited, indicating that Hsp70 must enter the dense phase of the nucleolus to interact with misfolded proteins and prevent potentially pathological interactions (Frottin et al., 2019). Yet, when cells are exposed to prolonged stress, or express pathogenic c9ALS/FTD-linked dipeptide repeat proteins (specifically, poly-PR), the nucleolus solidifies, suggesting that the protective power of Hsp70 in the nucleolus is limited, and extended insults to the nucleolus may contribute to neurodegeneration (Aladesuyi Arogundade et al., 2021; Frottin et al., 2019).

Leveraging Hsp110, Hsp70 and Hsp40 disaggregase activity as a therapeutic strategy is a delicate task. Not only are there multiple components to consider, but there are many members in each family of Hsp110, Hsp70 and Hsp40 proteins, which work together with varying degrees of efficacy (Nillegoda et al., 2017). Moreover, inducing this system at a level that enhances toxic fibril fragmentation but does not eliminate the fibrils may enhance protein-misfolding cascades and exacerbate toxicity (Tittelmeier et al., 2020). Therefore, a treatment that relies on this disaggregase machinery must hit a working range in which amyloid dissolution dominates over disease-amplifying fibril fragmentation. For example, conditions that promote removal of monomers from fibril ends rather than fibril fragmentation would dissolve amyloid without creating any new fibril ends, thereby limiting the number of sites available for further conformational replication (Duennwald et al., 2012).

## Skd3

In addition to cytoplasmic and nuclear protein disaggregases, humans also have organelle-specific ones. One example is the mitochondrial protein disaggregase Skd3 (also known as CLPB; Fig. 1D) (Cupo and Shorter, 2020). Skd3 is an AAA+ protein containing an ankyrin-repeat domain and a nucleotide-binding domain (NBD) that is homologous to Hsp104 NBD2 (Cupo and Shorter, 2020; Périer et al., 1995; Torrente and Shorter, 2013). *In vitro*, Skd3 can disaggregate model substrates, including α-synuclein fibrils, without assistance from chaperones like Hsp70 and Hsp40 (Cupo and Shorter, 2020). Skd3 disaggregase activity appears to be regulated by PARL, a mitochondrial inner-membrane protease. *In vitro*, mature-length Skd3 displayed only modest disaggregase activity when compared to Hsp104, but Skd3 lacking the residues upstream of the PARL cleavage site (_PARL_Skd3) displayed approximately fivefold higher disaggregase activity than Hsp104 (Cupo and Shorter, 2020).

In human cells, deletion of Skd3 leads to the aggregation of mitochondrial proteins associated with many essential biological processes, including respiratory-chain complex assembly and the cellular response to oxygen deprivation (Cupo and Shorter, 2020). Given the important role of Skd3 in maintaining the solubility of proteins involved in mitochondrial function, it is clear why Skd3 mutations can result in an aggressive mitochondrial disorder, 3-methylglutaconic aciduria, type VII (MGCA7; OMIM 616271) (Pronicka et al., 2017). Patients with MGCA7 experience nervous system deterioration, low white blood cell count and other motor, neurological and immune system deficits (Pronicka et al., 2017). Biochemical characterization of MGCA7-linked Skd3 variants revealed a strong inverse correlation between MGCA7 severity and Skd3 disaggregase activity (Cupo and Shorter, 2020). Thus, enhancing or restoring Skd3 disaggregase activity may be a treatment strategy for MGCA7 patients (Cupo and Shorter, 2020).

Skd3 is also an important target in acute myeloid leukemia (AML), in which its activity promotes drug resistance and cancer cell survival (Chen et al., 2019). Certain AML therapeutics, such as venetoclax, target mitochondrial apoptotic pathways, but patients often develop resistance to these drugs. Skd3 acts as a chaperone for several antiapoptotic factors in mitochondria, including OPA1 and HAX1 (Cupo and Shorter, 2020). Therefore, excessive Skd3 chaperone activity might antagonize venetoclax treatment (Chen et al., 2019). Indeed, Skd3 protein levels are higher in AML cell lines that are resistant to venetoclax than in venetoclax-sensitive cells (Chen et al., 2019). Additionally, Skd3 depletion in venetoclax-resistant AML cell lines and animal models increased the efficacy of venetoclax, confirming that venetoclax resistance is facilitated by Skd3 function (Chen et al., 2019). Thus, Skd3 is an unexplored therapeutic target for both cancer and protein-misfolding diseases.

## Karyopherins

Recent work has uncovered that nuclear-import receptors (NIRs) such as Karyopherin-β2 (Kapβ2; also known as TNPO1) can also antagonize aberrant phase transitions of specific disease-associated proteins (Fig. 1E) (Guo et al., 2019; Springhower et al., 2020). Kapβ2 recognizes cargo bearing a proline–tyrosine (PY)-nuclear localization signal (NLS), including the RBPs FUS, TAF15, EWSR1, HNRNPA1 and HNRNPA2 (Guo et al., 2019; Springhower et al., 2020). Remarkably, Kapβ2 can prevent and reverse condensation and fibrillization of these RBPs in the absence of ATP or any additional co-factors (Guo et al., 2018; Hofweber et al., 2018; Niaki et al., 2020; Qamar et al., 2018; Rhine et al., 2020; Yoshizawa et al., 2018). Likewise, importin-α proteins (also known as KPNAs) and Kapβ1 (also known as KPNB1), which recognize classical NLSs, can cooperate to prevent and reverse TDP-43 condensation and fibrillization (Chou et al., 2018; Guo et al., 2018; Hutten et al., 2020).

Importantly, these NIR activities are cytoprotective *in vivo*, as upregulation of Kapβ2 can prevent motor neuron degeneration elicited by ALS-linked FUS and muscle degeneration conferred by disease-linked HNRNPA2 (Guo et al., 2018). However, in disease, NIRs ultimately become overwhelmed and fail to counter pathological phase separation. In some cases, protein cargo bear disease-linked mutations in the NLS, as with FUS^P525L^, in which the proline residue of the PY-NLS is mutated, or in FUS^R495X^, in which the PY-NLS is largely deleted (Guo et al., 2019). These mutant forms of FUS are associated with very aggressive forms of early-onset ALS, and are more refractory to Kapβ2 activity (Gonzalez et al., 2021; Guo et al., 2018). In sporadic ALS and other neurodegenerative diseases for which there is no clear genetic component, NIR levels may decline due to stress and aging or otherwise become less effective, as defects in nucleocytoplasmic transport are a key feature of many neurodegenerative diseases (Guo et al., 2019; Kim and Taylor, 2017). Thus, finding ways to pharmacologically or therapeutically enhance NIR expression or activity or promote their interactions with cargo are promising strategies that are being actively developed.

## Tripartite motif proteins (TRIMs)

TRIMs represent another class of newly recognized ATP-independent human protein disaggregases (Fig. 1F). They are defined by the presence of a TRIM/RBCC motif comprised of a RING domain, one or two B-boxes, and a coiled-coil region (Zhang et al., 2020). The RING domain can act as an E3 ubiquitin or SUMO ligase to mark misfolded proteins for degradation (Williams et al., 2019). TRIMs are exclusively metazoan, with over 70 distinct variants in humans (Zhang et al., 2020). Remarkably, TRIM11 abrogates the aggregation of diverse proteins and solubilizes preformed aggregates, including neurotoxic amyloids formed by α-synuclein and polyQ (Zhu et al., 2020). These activities can be separated from TRIM11 SUMO-ligase activity, but, when combined, they can promote the degradation of misfolded proteins (Zhu et al., 2020). Importantly, TRIM11 dissolves α-synuclein fibrils *in vitro* and suppresses α-synuclein toxicity in cell and mouse models of PD. Indeed, intracranial delivery of adeno-associated viruses expressing TRIM11 suppressed prion-like spread of α-synuclein pathology, neurodegeneration and motor impairments in a PD mouse model (Zhu et al., 2020). The ability of TRIM11 to mitigate these phenotypes indicates that TRIM11 does not release toxic α-synuclein species, unlike some circumstances for the previously discussed Hsp110, Hsp70 and Hsp40 system (Tittelmeier et al., 2020).

Other TRIMs can also function as ATP-independent chaperones and disaggregases, including TRIM19 (also known as promyelocytic leukemia protein) and TRIM21 (Guo et al., 2014; Zhu et al., 2020). In the nucleus, TRIM19 facilitates the degradation of an insoluble variant of ataxin-1 with an expanded polyQ repeat, which is linked to spinocerebellar ataxia (Guo et al., 2014). TRIM19 also promotes the solubilization and degradation of other aggregated proteins, including polyQ-expanded huntingtin and TDP-43 (Guo et al., 2014). Although care must be taken to prevent excessive off-target sumoylation and ubiquitylation by TRIM proteins, further exploring and enhancing the activities of TRIM family proteins is an exciting new avenue for combating aberrant protein states.

## RNA as a chaperone

RBPs with PrLDs aggregate in several neurodegenerative diseases, and these aggregated structures may be devoid of RNA (Mann et al., 2019). This absence of RNA from these aggregates indicates that RNA itself may promote RBP solubility. Indeed, injecting RNase into the nuclei of cells causes rapid nuclear condensation of several disease-linked RBPs, including FUS, EWSR1, TDP-43 and TAF15 (Maharana et al., 2018). Furthermore, RBPs bearing mutations that abrogate RNA binding aggregate more readily in cellular models (Mann et al., 2019). Thus, specific RNAs might act as chaperones for their RBP partners. For example, translation of *TDP-43* mRNA is regulated by TDP-43 binding to the 3′ untranslated region of its own mRNA, and this untranslated sequence was identified as a candidate oligochaperone (Ayala et al., 2011; Sun et al., 2014). In particular, a 34-nucleotide sequence (Clip_34nt) in the 3′ untranslated region of the *TDP-43* transcript binds to TDP-43 with high affinity, which could be employed to antagonize TDP-43 aggregation (Fig. 1G) (Ayala et al., 2011; Mann et al., 2019; Sun et al., 2014). To test this notion in neurons, Mann and colleagues developed an optoTDP43 system in which TDP-43 is tagged with a Cry2 domain that oligomerizes under blue light (Mann et al., 2019). Neurons expressing optoTDP43 form cytoplasmic TDP-43 inclusions as a function of blue light exposure, and these inclusions are toxic, even in the absence of any external stressor. However, treating neurons with Clip_34nt before inducing TDP-43 aggregates significantly improved neuronal survival, whereas a scrambled Clip_34nt sequence had no protective effect (Mann et al., 2019). This discovery demonstrates that specific ‘bait’ RNAs might be developed to mitigate toxic RBP conformations in disease (Portz et al., 2021).

## Outlook

The research advances outlined in this article demonstrate that significant strides have been made in both our understanding of diseases connected with aberrant phase transitions and how we might treat them. Now, the next step is to apply this knowledge to more complex animal and human systems. One approach would be to use adeno-associated viral vectors to deliver genes that encode potentiated disaggregases into mouse models and ultimately patients (Tariq et al., 2019). Other strategies for gene delivery might also be possible, including lipid-containing nanoparticle-mediated delivery of chemically modified mRNAs to the central nervous system akin to the technology that has enabled mRNA vaccines (Kormann et al., 2011; Pardi and Weissman, 2020; Shorter, 2016). Notably, for delivery of RNA-based chaperones, there are several examples of US Food and Drug Administration (FDA)-approved drugs based on the direct delivery of antisense oligonucleotides to cells, and these could be adapted for the delivery of short protective RNAs to combat RBP misfolding (Bennett et al., 2021; Leavitt and Tabrizi, 2020; Portz et al., 2021).

The development of novel tools to treat neurodegenerative diseases is encouraging, yet a persisting major challenge is early diagnosis. Often, when a patient begins to display symptoms of neurodegeneration, extensive protein misfolding and neuronal death have already occurred. Here, disaggregase-based approaches are particularly attractive because they can act on aggregates and toxic oligomers even after they have formed (Shorter, 2008, 2016, 2017). These strategies to rescue degenerating neurons might even be combined with cell-reprogramming strategies that convert glia to neurons and thereby replenish neurons that have already been lost to re-establish neural circuits (Qian et al., 2020; Zhou et al., 2020). Diagnostic methods are evolving, though, and recent efforts to identify biomarkers for neurodegenerative diseases have been successful (Jeromin and Bowser, 2017). Thus, in the future, we may be able to diagnose patients in the early pre-symptomatic stages of their disease and apply disaggregase-based therapies, which could both stop and reverse the formation of pathological protein conformations, thus protecting neurons and preventing cognitive and motor decline.
